# Address before the Lowndes County (Miss.) Medical Society, at the Anniversary Meeting, May 29, 1873

**Published:** 1873-09

**Authors:** J. W. M. Shattuck, W. L. Lipscomb


### SOUTHERN MEDICAL RECORD.

VOL. III.—SEPTEMBER, 1873.—No. 9.

### PART I.

### ORIGINAL COMMUNICATIONS.

### ADDRESS BEFORE THE LOWNDES COUNTY (MISS.) MEDICAL SOCIETY, AT THE ANNIVERSARY MEETING, MAY 29, 1873.

BY DRS. J. W. M. SHATTUCK AND W. L. LIPSCOMB.

[*Concluded.*]

Well may the hand of exclamation be lifted, and the mind stagger in its contemplation.

Think of the vastness of its *knowledge*. Beginning with the human body—with two hundred and forty bones, four hundred muscles; with its veins and arteries, and nerves, and vessels, and fluids, and organs; with hair, and nails, and skin—medicine has studied the number and the shape, and the weight and the color, and the density and the structure and connection of each one of them with an accuracy that includes the minutest atom of their composition, and with a description so perfect that the smallest points and lines, and the faintest shades of color are accurately delineated.

Nor is this all. It goes back to the first cell from which the human body is formed, and marks and notices and numbers the growth and development, and change and structure of all these parts of the human body, in the fœtal state, before immergence from the maternal womb, during infancy, and youth to perfect manhood.

Nor is this all. Just as exactly and accurately as these parts have been studied in *health*, so has been the work all repeated in *disease.* Not satisfied yet! All these parts are studied with reference to *function* and *use* in the human economy when in a state of health; and, yet once again, when these uses and functions are perverted in disease.

Changing the view from the human body to the *race* stand-point, and we see medicine standing at the accouchment, and, like a great foster-mother, receiving the breathing, pulsating mass of infancy in her kind arms; and benignantly taking her seat beside the cradle, she sits the presiding genius of the nursery. Medicine has proven herself to be Childhood's kindest and most useful friend. Time was when "children were looked upon (apparently) as destitute of the ordinary qualities of our nature—as if they were hardly human beings. Their education was shamefully mismanaged, and their diseases, ill understood, were treated in the most harsh, cruel and unscientific manner. Over-dosing, ill ventilation, under-feeding were the order of the day." Medicine, reversing all this, sees in the child the lineaments and capabilities of the future man, and has bestowed upon his wants and diseases her maturest study and most patient toil.

Stepping out of the nursery, medicine enters the school-room and becomes joint occupant with the teacher upon his rostrum, and, with wise oversight, sees that our worked brains, stooped shoulders, pallid faces, weak eyes, impaired constitutions, are not the necessary concomitants of a liberal education. Youth is the time for physical growth and the perfection of the senses. Hence, medicine has shortened its hours of mental labor, built gymnasiums, devised amusements, and led the young into a field of development in accord with the designs of Nature.

Medicine is the great counsellor and friend of Manhood. Without health, what is man? A *sick man* is more the object of pity than a tender babe or decripid age. Disease stretches him upon his bed; soon strength is all gone, and the Hercules of to-day is too weak to rise; a woman lifts him up; a little child hands him his cup of water. Poor, pitiable object of compassion, he receives assistance from the most menial and degrading character! He frets, he whines, he cries; offensive odors exhale from his diseased-stricken body. How men ought to love the doctors! What fostering care medicine, in all her departments, ought to receive at their hands!

And has medicine done nothing for woman? Yes! *all she could;* and yet your speaker dare not parade a single achievement, present the remembrance of a single benefit, or recite her story of toil and study. And why? Woman has already returned to medicine all she ever received, and the rich argosies of her gratitude hide every benefit bestowed upon her.

Obligation hangs like a weight on the side of medicine; and in her presence, medicine confesses herself dumb to express, and bankrupt to repay a tithe of her indebtedness.

Woman has cheered, with her presence, the midnight vigils of the physician, as he sat by the bedside of the sick and the dying. Her words of sympathy have encouraged him when the gloomy pall of disappointment and failure mantled his spirit; her deeds of kindness have supported his sinking frame, as it bent, exhausted with fatiguing labor; her gratitude, as it overflowed from the depths of her tearful eyes, have been to him richer rewards than glittering gold—more precious than the laurel wreaths of fame. An angel of mercy, she has ministered alike to physician and patient; an untiring nurse, she has prepared all his medicine and added her soothing voice to the letheons which she gave. Her words of hope have strengthened more than the cups of wine, and the magic of her soft, cool hand has quieted the fever that raged in the blood. In the sick room, in the wards of the hospital, in the track of the pestilence beside the bloody streams of the battle-field, she has been the steadfast ally and his faithful friend.

And now, medicine, with all her laurels on her brow, and every star and badge glittering on her breast, holding in her hand the wand of victory, surrounded by trophies from ten thousand fields, and attended by a vast retinue of obsequious sister sciences, bows on bended knee in woman's presence, and asks, as her highest honor, that she may but kiss the hem of her garment.

The science of medicine includes a knowledge of almost the entire physical world. Every plant, and herb, and tree, with their roots, and bark, and leaves, and seed; every mineral and metal; earth, air, fire and water, animal and insect, have been objects of her close and scrutinizing analysis. She has laid her hand upon the whole material of physical nature, and the extent of her researches are bounded only by the limits of the Universe. Medicine studies every other science—Chemistry, Geology, Zoology and Botany, Natural History and Natural Philosophy, Agriculture and Horticulture, Moral Science and Mental Science, are all parts of her great acquirements. Government and society are portions of her well-explored territory. She enters the halls of legislation and enacts sanitary laws, and regulates the public hygiene; she examines the great problems of population, and limits and extends the duration of human life; she builds hospitals and asylums, legislates for the insane, and the blind, and the inebriate; she takes her place on the staff of the general and goes out to war, and in camp and battle-field spreads her stores of virtue and accomplishes her feats of daring, courage and splendid success; she gathers her instruments and her books, and takes charge of the exploring ship and pushes her investigations into the arctic regions of the North, and finds her way into the boundless jungles of torrid Africa. Medicine is the handmaid of society: she presides at the table and in the dressing-room; she is her architect and gardener; she regulates the selections of Hymen, and holds a warning finger over the lasciviousness of Cupid. In all that pertains to the family or the individual, medicine has studied and developed laws for the protection and increase of their health and happiness.

But we tire in enumeration of the vastness and extent of medical science; we must even forego a picture of the labor and intellectual force that was expended in accomplishment of such grand results, and leave untold that thrilling story of moral heroism and physical courage and exhaustless self-denial that characterized her army of heroes, as amid the breath of the pestilence and the bloody waves of war, in the haunts of disease and in the charnal houses of death, they accomplished feats and achieved success, before which the warrior pales and the brave soldier makes obeisance.

The physician who, with an intelligent appreciation of the duty, and the labor, and the danger before him, sets out in the study and practice of medicine, is a theme for the orator and an inspiration for the poet. He who, with forethought, cuts the cords that bind him to wife, and children, and home, leaves the pleasures of society, foregoes the calls of ambition, renounces wealth and honor, and choses the solitary path of labor, and toil, and danger, among the solitary hovels of the poor, amid the ravages of disease and by the cold streams of death, and who, after a life of exhaustion and premature decay, is content to look back upon a career of abnegation and crucifixion of self, is the world's anomaly, and should be the world's hero.

Medicine is no man-science; its profession no place of ease; its practice no lucrative sinecure. The world has secured the benefit of her labors, and makes large demands upon her laborers; but it has been niggard in reward, and penurious her evidences of appreciation. If money, or honor, or distinction had been the aim of physicians, they never would have chosen medicine as a profession. But they make no complaint. Thanks to the enobling character of the profession, and the sustaining principles upon which it is based, they have learned that "virtue is its own reward." Integrity and usefulness are jewels more valuable than money or place, and they prefer the respect of merit to the gifts of favor.

Being human, physicians are sometimes disturbed by the hypercriticisms of the world upon the plans and details of their labor—especially when these ungenerous thrusts are made at the conventional rules and utilitarian regulations by which, as a profession, they are governed.

Physicians, as a class, like every other class of men, have what has become to them *a code of manners—a system of medical ethics*—that certain rule of politeness—of good breeding—of high courtesy, as simple as the salutations of the morning or the manners of the thoroughfare, and as pure and free from evil as the moral law. Medical ethics is the crystalization of the morality, and charity, and refinement of the profession, and is as indispensable a part of a medical gentleman as the purity of his own character and the inbred instincts of his own refined nature. An educated physician can no more violate the regulations of his code than a polite gentleman can trample upon the rules of society. Medical ethics is no Procrustean bed to amputate and trim the profession into the same shape and mould; it is not a register of the oaths of a secret, clandestine association of public enemies; it is not the regulations of a "*ring*" of public plunderers, nor a concoction of selfish means for the advancement of a set of arrogant bigots or greedy cormorants who feed and fatten upon the public welfare. Medical ethics is the oil that lubricates the friction of a great philanthropic profession, and keeps in unison and harmony the parts of a complex whole. It is the golden rule of the science. Thou shalt love medicine with all thy soul, and mind, and strength, and thy brother physicians as thyself. Chesterfield is a volume beside it, and the decalogue more numerous and less lucid in its requirements. Its grand design was to root selfishness and meanness out of the profession, and to separate medicine from the world of barter and trade, and to preserve its own inherent philanthropic and disinterested standards of action.

Medicine does, in its code of ethics, refuse to enter the arena of money competition and to be subject to the rules of purchase and sale—of speculation and lottery. The medical fee-bill of a society, or the printed standard of charges for service by the regular faculty of a community, is one of those simple outgrowths of professional liberality by which they offer protection to the community from the exhorbitant charges of some blatant, advertising pretender, or from the imposition of the pinchbeck, shoddy acquirements of some *cheap* doctor. Organize medicine, to-day, upon the basis of trade, turn loose all the devil, and Yankee, and Jew there is in the doctors, and in one twelve-month they would bankrupt California and the Indies, and the plethora of their pretense would exceed the repletion of the public treasury. The world would have on its hands the care of the countless of poor, who could not buy; and the rich, upon the payment of their bills, would have no money left for charity. Who are the rich men of your cities? Are they the regular faculty of medicine? No, sir; they are men who trade in human health, and sell medicine according to the chances and circumstances that surround the victims on whom they operate. The credulity of the sick, the ignorance of the multitude, and their own sordid souls, constitute their stock in trade. Regular medicine has no secret nostrums to sell; no patented inventions to protect; no universal theories to teach; no universal catholicans to cure; no blazing placards to advertise. Among the regular faculty, there are no professional cut-throats, nor invidious detractors. Paraphrasing the tenets of our own noble code, we shout—

> "Or, if no basis bear my rising name
> But the fallen ruins of a brother's fame,
> Then teach me, Heaven, to scorn the guilty bays;
> Drive from my heart that wretched lust of praise.
> Unblemished let me live, or die unknown;
> Grant me an honest name, or grant me none."

Medicine, like chastity, blushes to defend herself, and weeps to find her white garments soiled by the contacts of pollution and filth.

Members of the Columbus and Lowndes County Medical Society: In the language of another, let me exhort you to remember, "that ours is a profession devoted exclusively to the welfare of mankind. Sordid and mercenary motives cannot be admitted. We must stand out as the benefactors of our race, and consecrate our time, our talents and our lives to the public weal. As guardians of true science and sound knowledge, let us beware of charlatanism, and discard the babbling of empiricism, though they come with both hands full of gold. It is one of the sad things of our fallen world and fallen nature, that men love to be deceived. You can deceive them, if you will, and fatten upon their follies. *You will not do it*. God entrusts to your keeping a nobler heritage. The possession of knowledge above your fellows is the very reason why you should not play upon their ignorance, but rather enlighten them and lead them to better things. Hold fast the sacred trust committed; never lower your standards; be true to your code and to each other. Better, by far, wait years for success, than to sacrifice your principles and sell your manhood for money; better die poor, with clean hands and a good conscience, than walk in marble halls or sleep in granite sacophagi."

We close by congratulating you upon the success of your last years work. By your elevated professional bearing and devotion to your duty, you have merited and received the confidence and appreciation of the community in which you live; at home and abroad, your names are written high on the professional roll, and your productions have been marked with the stamp of highest merit,

Some of you have held positions of responsibility and the gravest official trust, and Columbus thanks you for your energy and success in driving the small-pox from her limits.

With a tear of remembrance for our brother whom the angel of death cut down at his post, and a sigh of regret at the absence of our much-loved and respected President, members of the Columbus and Lowndes County Medical Society, I bid you God-speed and good-night!

NOTE.—The above address is the joint production of Drs. J. W. M. Shattuck and W. L. Lipscomb. Dr. Shattuck was the retiring President, and the duty of the annual address devolved upon him. But after the selection of his subject and the collection of his material, he was taken sick, and compelled to leave home before its completion. His material and manuscripts were turned over, by order of the Society, to Dr. Lipscomb, with instructions to prepare and deliver the address at their anniversary meeting; which duty was performed, and the address delivered by Dr. Lipscomb, as above written.

*Columbus, Mississippi, April* 29, 1873.

